# Ethambutol-Induced Optic Neuritis and Vision Loss: A Case Report

**DOI:** 10.7759/cureus.64873

**Published:** 2024-07-18

**Authors:** Jaykumar Patel, Chetna Patel, Akash Shah, Paras Shah, Sajal Pandya, Brijesh Sojitra

**Affiliations:** 1 Pharmacology, Government Medical College, Surat, Surat, IND; 2 Pharmacology, Government Medical College and New Civil Hospital, Surat, Surat, IND; 3 Pharmacology and Therapeutics, Government Medical College, Surat, Surat, IND; 4 Pharmacology, Government Medical College Surat, Surat, IND

**Keywords:** vision loss, side effect, anti-tubercular treatment, optic neuritis, ethambutol

## Abstract

Ethambutol is a first-line chemotherapeutic agent, which is commonly used in combination with other drugs for the treatment of tuberculosis. Ethambutol-induced optic neuritis is a serious and rare side effect that is either dose or duration-related and causes progressive painless vision loss, and cecocentral scotomas in the visual field. A rare case of ethambutol-induced optic neuritis was reported in a 52-year-old female who was taking anti-tubercular treatment for pulmonary tuberculosis for five months. She presented with painless diminished vision in both eyes. The patient was diagnosed with a rare case of optic neuritis through various examination methods. Ethambutol was stopped and therapy was continued with oral prednisone, zinc, and vitamin B complex being started along with anti-TB treatment. She showed no marked improvement in visual parameters until the last follow-up. The patient died due to cardiopulmonary arrest as a consequence of pulmonary tuberculosis.

## Introduction

Ethambutol is a first-line chemotherapeutic agent used along with isoniazid, rifampicin, and pyrazinamide, for pulmonary tuberculosis [1,2]. Ethambutol inhibits the enzyme arabinosyl transferase, which is important for synthesising the mycobacterial cell wall. Previously, each drug had been prescribed separately based on the patient’s weight [2]. According to the National Tuberculosis Elimination Program (NTEP) guidelines, all cases that are sensitive to first-line drugs should be treated for a two-month intensive phase and a four-month continuation phase [1].

Now anti-tubercular drugs are given in the form of fixed drug combinations (FDCs) as per the patient’s weight [2]. Ethambutol has shown better patient compliance due to good acceptability and better tolerance as it has minimal adverse effects. Adverse effects associated with ethambutol are rash, pruritis, joint pain, optic neuritis, gastrointestinal (GI) upset, malaise, headache, dizziness, mental confusion, disorientation vision loss, and visual field defect [3]. A small percentage, ranging from 1% to 2%, of patients administered ethambutol may experience Ethambutol-Induced Optic Neuritis [4]. Every year, around 9.2 million new cases of TB are reported worldwide and nearly 100,000 patients develop toxic optic neuropathy due to ethambutol treatment [4].

## Case presentation

**Figure 1:**
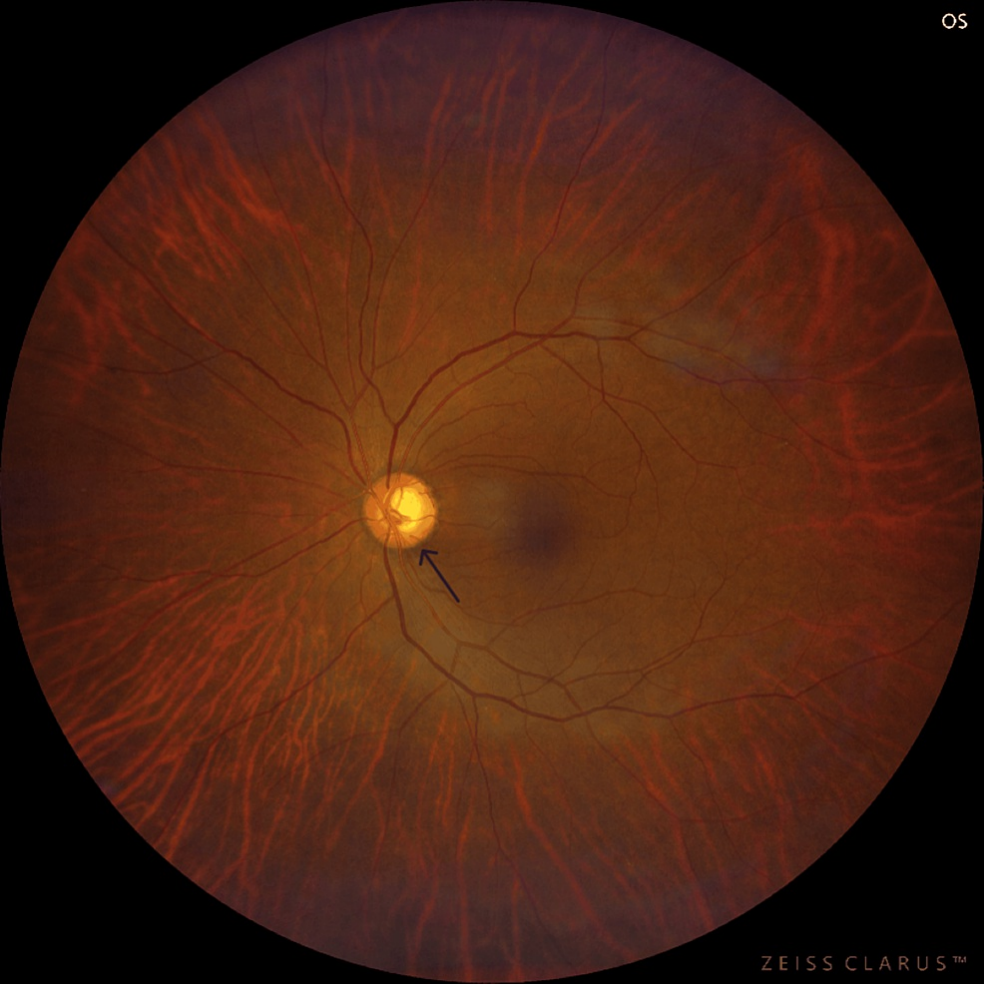
The left-eye fundus examination image at the time of the presentation The fundus examination showed disc hyperaemia and blurred disc margins in the left eye.

**Figure 2:**
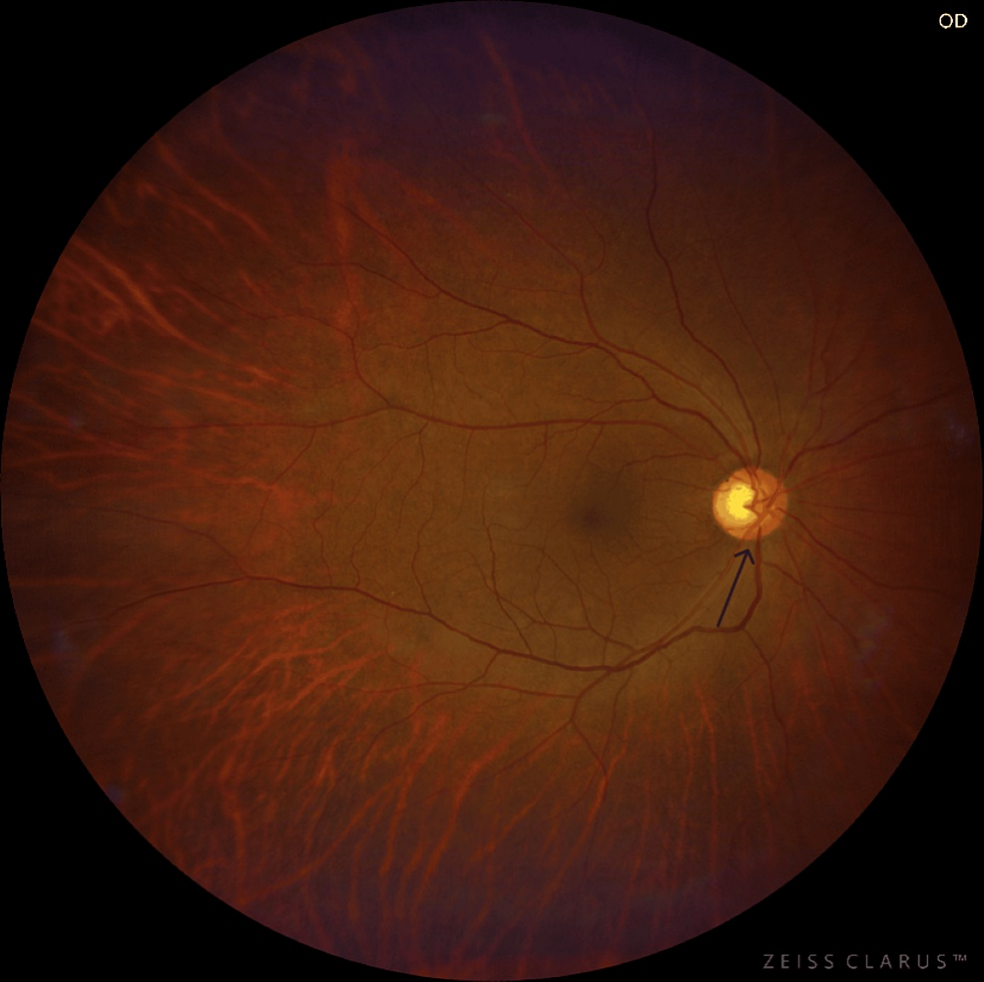
The right-eye fundus examination at the time of the presentation The fundus examination showed disc hyperaemia and blurred disc margins in the right eye.

A 52-year-old female weighing 55 kg was on FDC (isoniazid, rifampicin, and pyrazinamide) four tablets/day, for pulmonary tuberculosis. After five months of treatment, she presented with bilateral progressive, painless vision impairment, along with tingling and numbness in both feet for the past three to four months. The symptoms started about three and a half months after beginning therapy but she ignored them. On examination, her BCVA (best-corrected visual acuity) was 1/2m counting fingers for the right eye and 6/36 for the left eye. IOP (intraocular pressure) was 16 mm Hg in the right eye and 14 mm Hg in the left eye through Goldmann Applanation Tonometry (GAT). There was no relative afferent pupillary defect (RAPD) and the pupil was normal. The periorbital area was normal, and the ocular movements were full and free. Color vision was affected by a greater impairment of red-green discrimination. The lids, adnexa, and conjunctiva were bilaterally normal, and the bilateral cornea was normal in size and shape. The anterior chamber was deep and quiet in both eyes. Iris of both eyes were normal in color and pattern. The bilateral lens had nuclear sclerosis grade 1. The fundus examination showed disc hyperemia and blurred disc margins in both eyes (L<R) (Figures 1, 2).

Ethambutol was stopped immediately, oral prednisone 40 mg, zinc (20 mg), and vitamin B complex was started along with other anti-TB treatments. She did not show any marked improvement in visual parameters after one month of follow-up, on examination, her BCVA (best-corrected visual acuity) was 1/2m counting fingers for the right eye and 6/36 for the left eye. IOP was 16 mm Hg in the right eye and 18 mm Hg in the left eye through GAT. After the patient died due to cardiopulmonary arrest. According to the WHO UMC causality assessment scale [5], it is categorized as possible. As per the Preventability Modified Schumock and Thornton scale, it was probably preventable [6].

## Discussion

Optic neuritis and vision loss are rare and severe side effects of ethambutol. Ethambutol-induced optic neuritis can result in loss of red-green color discrimination, decreased visual acuity, and visual field defects [4]. Studies have indicated that ethambutol-induced optic neuropathy (EON) is an adverse effect that depends on both the dosage and duration of treatment [4]. The probability of this reaction is higher with greater doses and longer use of ethambutol. This happens in 15% of patients who take 50 mg/kg daily, 5% who administer 25 mg/kg daily, and below 1% of patients on a daily dose of 15 mg/kg [3].

Since ocular toxicity can occur at any dose of ethambutol, there is no predetermined safe dose. Also, the onset time of optic neuritis cannot be anticipated. Usually, it starts 4 to 12 months after therapy begins, but it might also happen soon after the onset of treatment. Adverse effects may be influenced by age, alcohol, smoking, renal status, treatment duration, and comorbidities. Patients are usually presented with gradual painless loss of vision. Initially, it can be noticed as reading-related blurring but may indicate central field abnormalities. There is bilateral involvement seen simultaneously, but the onset might also be unilateral. Affected central fibers hamper the visual acuity on usual presentation, but peripheral fibers can also be affected rarely with peripheral field constriction. In turn, it may progress to optic atrophy, which appears as temporal disc pallor when a fundoscopic examination is performed [7,8].

Optic neuritis caused by ethambutol is usually reversible after discontinuation of medication, but recovery is time-consuming [3]. It is unknown how ethambutol causes optic neuritis, although theories have been put forward. One such theory is based on the fact that ethambutol and its metabolites can chelate zinc, causing a disturbance in retinal homeostasis [7,8]. In addition, ethambutol is believed to interrupt oxidative phosphorylation by acting on iron-containing complex I and copper-containing complex IV, leading to reactive oxygen species production which may cause injury to the retinal ganglion cells [7]. Zinc and copper deficiencies, caused by the metal-chelating effects of ethambutol, are believed to contribute to the development of neuropathy similarly deficiencies in vitamins such as E, B1, B9, and B12 may worsen optic neuritis, To mitigate this risk, supplementing these minerals and multivitamins has been suggested [9].

To develop tolerance and reduce adverse reactions, desensitisation is exposing the immune system to increasing dosages of an allergen or drug over time. For ethambutol-induced optic neuropathy, this process may include the careful reintroduction of ethambutol at lower doses and increase the dose gradually. The goal is to reduce the risk of optic neuropathy while allowing the patient to continue benefiting from ethambutol’s antitubercular effects.

In this case, the patient was on anti-tubercular treatment as per NTEP guidelines and was presented with defects in visual acuity, colour vision, and visual field defects. The total dose of ethambutol had not exceeded the upper limit of 25 mg/kg or a daily dose of 1500 mg, but there are no safe dose in ethambutol. After stopping ethambutol, the patient was treated with oral prednisolone, zinc, and vitamin B complex. However, the patient showed no significant improvement in visual defect, probably due to her age indicating that the recovery is very slow and time-consuming. According to the World Health Organization-Uppsala Monitoring Centre (WHO-UMC) causality assessment scale, it is categorized as possible, indicating that there is a suspected association between the ethambutol drug and optic neuritis. and as per the Preventability Modified Schumock and Thornton scale, it was probably preventable indicating that optic neuritis could likely have been avoided with preventive measure or different management.

## Conclusions

Prompt diagnosis, discontinuation of ethambutol, early treatment, and continued eye tests can reverse this situation and improve the patient’s vision. Patients with risk factors who are treated with anti-tubercular treatment (ATT) should undergo a pre-treatment ophthalmological examination and be given the adjusted dose according to the risk factors. Educate the patient to identify symptoms and report as early as possible.
